# Molecular SPECT Imaging: An Overview

**DOI:** 10.1155/2011/796025

**Published:** 2011-04-05

**Authors:** Magdy M. Khalil, Jordi L. Tremoleda, Tamer B. Bayomy, Willy Gsell

**Affiliations:** ^1^Biological Imaging Centre, MRC Clinical Sciences Centre, Imperial College School of Medicine, Hammersmith Hospital Campus, Du Cane Road, London W12 0NN, UK; ^2^Nuclear Medicine Section, Medical Imaging Department, King Fahad Specialist Hospital, P.O. Box 11757, Dammam 31463, Saudi Arabia

## Abstract

Molecular imaging has witnessed a tremendous change over the last decade. Growing interest and emphasis are placed on this specialized technology represented by developing new scanners, pharmaceutical drugs, diagnostic agents, new therapeutic regimens, and ultimately, significant improvement of patient health care. Single photon emission computed tomography (SPECT) and positron emission tomography (PET) have their signature on paving the way to molecular diagnostics and personalized medicine. The former will be the topic of the current paper where the authors address the current position of the molecular SPECT imaging among other imaging techniques, describing strengths and weaknesses, differences between SPECT and PET, and focusing on different SPECT designs and detection systems. Radiopharmaceutical compounds of clinical as well-preclinical interest have also been reviewed. Moreover, the last section covers several application, of *μ*SPECT imaging in many areas of disease detection and diagnosis.

## 1. Introduction

Small animal imaging has become an integral part of molecular medicine. Translation of ideas from bench to the clinic needs a verification and validation step where molecular diagnostic modalities are substantial tools in developing new tracers, drug design and therapeutic regimens. In the last few years, there was a tremendous change and focus on the development of new microscale imaging systems of spatial resolution and detection sensitivity that relatively cope with the requirements of imaging small animals such as mice and rats. The focus was not only on instrumentation but also was accompanied by contrast agents/probes/biomarkers that target specific biological processes. Similarly, as these molecular agents are developed to suit particular biochemical targets, there are corresponding imaging techniques able to detect this particular signal. There is a relatively large array of imaging modalities that have their individual characteristics. The molecular imaging arena has been revolutionized also by hybridization/fusion of these techniques into single imaging devices. The best example that can be drawn from the literature as well as from the clinical practice is the recent implementation of PET/CT and (SPECT/CT) in clinical oncology and other important areas of disease detection. Apparent PET/MRI implementation and direct incorporation in routine practice is still controversial and active research is underway and its spread in the market will be determined in the near future [1].

However, there are emerging promising approaches that are undergoing extensive research work and investigation (with some successful results) for possible translation into the clinic. These include contrast-enhanced molecular ultrasound with molecularly targeted contrast microbubbles, optical imaging with fluorescent molecular probes, Raman spectroscopy/microscopy and more recently, photoacoustic imaging; a hybrid optical and ultrasound technique (see [2–4] for review).

Nuclear medicine has an established role in this context and tomographic tools such as single photon emission computer tomography (SPECT) and Positron Emission Tomography (PET) have their significant contribution to the world of molecular imaging [4, 5]. However, anatomical techniques such as CT and MRI through their high spatial resolution capabilities serve to identify morphological changes in small structures. When these imaging modalities are combined in one imaging session, the amount of information obtained can synergically and significantly improve the diagnostic process and its outcome when compared to a single diagnostic technique [6]. 

Another important aspect of preclinical imaging is the ability to study the physiology over several time points referred to as longitudinal studies. A significant reduction of cost, number of animals as well as reduction of intervariability among subjects are among the most important outcomes of this technology. Thus, one can avoid animal dissection, *ex vivo* tissue counting and other autoradiographic studies.

## 2. Instrumentation

### 2.1. Strengths and Weaknesses

Diagnostic modalities can generally be distinguished based on whether they are structural or functional imaging techniques. Computerized tomography (CT) and magnetic resonance imaging (MRI) are well-known diagnostic tools that provide very high structural information of the tissue under investigation when compared to functional techniques such as SPECT or PET. MRI provides better soft tissue contrast even in absence of contrast media, a feature that is absent in CT scanning. Ultrasound procedures use high-frequency ultrasound waves to differentiate between different anatomical structures and safe (radiationless) diagnostic imaging technique. However, it has less functional or physiological significance when compared to nuclear modalities. Optical imaging such as bioluminescence and fluorescence are also functional modalities, but their limited spatial resolution, limited penetration capabilities and other factors contribute to their unease of transition to clinical practice [3]. 

 The relative weaknesses and strengths that exist among imaging techniques are important to be understood. One can notice that the spatial resolution of MRI and CT is significantly higher than that of SPECT and PET. However, the detection sensitivity of SPECT and PET is significantly higher than those given by structural modalities and moreover can detect tracer concentration in the picomolar or nanomolar range. Both approaches use the tracer principal to detect physiological abnormality or disturbed biochemical process. 

 The key elements in radionuclide imaging are a biomarker and an imaging device. The first should have high specific, as well as sensitive characteristics to optimally study a molecular or cellular phenomenon. The imaging device is a radiation detector with specific performance to localize activity distribution within the human body or the animal. The most commonly used instrument in SPECT imaging is the conventional gamma camera that was invented in the middle of the last century by Anger [7]. However, for detection of coincidence events and localization of PET-administered compounds, a PET scanner is normally used. Both imaging devices have witnessed a significant change in the last decade in terms of performance characteristics as well as diagnostic quality. 

On the other hand, MRI techniques do not relay on ionizing radiation, and thus, it is one of the features that characterize magnetic resonance procedures over other methods. Because of these inherent differences, there has been a large interest to combine more than one or two modalities into one imaging system able to morphologically and functionally address pathophysiologic questions. The present review will generally discuss many aspects of small animal micro-SPECT (*μ*SPECT) imaging including instrumentation, molecular imaging probes used in preclinical and clinical practice, and the last section will cover some important and valuable preclinical applications. Before this discussion, the author would like to outline some major differences that exist between SPECT and PET imaging.

### 2.2. SPECT versus PET

In clinical practice, almost all nuclear medicine procedures that use single photon emission tracers rely on the use of the gamma camera. It is a gamma ray position sensitive detector that typically consists of large slab of scintillator crystal with position circuitry and energy determination. To localize the emission site of the released photons, a multihole collimator is mounted on the front face of the system to provide a spatial correlation of the detected events.

 Hal Anger introduced the gamma camera as a novel detection technique able to localize an activity distribution of an administered radionuclide. However, his original prototype in 1953 was a camera in which a photographic X-ray film was in contact with NaI(Tl) intensifying screen. He used a *pinhole collimation* and small detector size to project the distribution of gamma rays onto the scintillation screen [7]. Initially, the camera was used to scan patients administered by therapeutic doses of 131-I. Disadvantages of this prototype were (1) small field of view of the imaging system (4 inch in diameter) and (2) poor image quality unless a high injected dose or long exposure time are applied. In 1958, Anger succeeded in developing the first efficient scintillation camera, and marked progress in the detection efficiency was realized by using an NaI(Tl) crystal, photomultiplier (PMT) tubes, and a larger field of view. 

**Figure 1:**
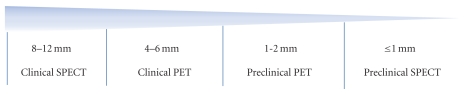
Spatial resolution across the clinical and preclinical SPECT and PET imaging scanners.

 Spatial resolution and detection sensitivity are two important performance characteristics that play an important role in molecular imaging research using SPECT and PET tracers. Although the clinical gamma camera can provide a tomographic resolution of about 10 mm, some preclinical SPECT scanners can provide a submillimeter spatial resolution pushing down to subhalf millimeters using a specialized dedicated multipinhole geometry [8]. This situation is different in clinical and preclinical PET imaging where the spatial resolution of preclinical PET scanners is about 1-2 mm while that of clinical PET scanners lies in the range of 4–6 mm. Dedicated brain PET scanners, however, can achieve a slightly better spatial resolution (*≈*2.5 mm) in the centre field of view. These resolution differences are mainly due to the fact that SPECT systems are not affected by some physical and fundamental limits that hinder the PET camera to reach sub-millimeter ranges although some research groups were able to achieve a resolution of less than 1 mm using fine segmented lutetium orthosilicate (LSO) crystal [9].  Figure 1 clearly defines the position of *μ*SPECT in the molecular imaging matrix.

 Many factors serve to impact the final reconstructed images of data acquired from a PET scanner. These are crystal size, positron range, photon acollinearity, intercrystal interaction and scatter, depth of interaction and the reconstruction algorithm. In preclinical PET machines, positron range appears to be the most important challenge that needs to be tackled to improve the spatial resolution of the PET images. However, the current generation of clinical PET scanners is slightly affected by the positron range, but correction of the phenomenon was shown to be effective in positron emitters of high maximum kinetic energy [10–12]. These issues are obviously absent in clinical as well as preclinical SPECT systems. The gamma camera relies on hardware collimators to determine the photons trajectory and hence able to localize the emission site by analyzing the electronic signal detected by the imaging detector. This hardware collimation plays a significant role in reducing the overall system sensitivity as well as the spatial resolution. 

 The intrinsic resolution of gamma camera is about 3-4 mm and tomographic SPECT acquisition reveals a spatial resolution, as mentioned above, not better than 10 mm. However, a new trend of designing semiconductor systems is emerging in the field, providing a significant improvement in spatial resolution, and other performance measures [13, 14].

SPECT degrading factors have been extensively studied in the literature and, namely, include attenuation, scatter and resolution effects, in addition to motion artifacts. Apart from the later, most of these physical issues can be resolved in great part by the use of SPECT/CT systems. These imaging degrading factors have a relatively smaller impact on the overall image quality in small animal imaging due to the smaller size of the rodents (mouse 20–40 g, rat 250–550 g) in comparison to standard human (75 kg). Nevertheless, correction for photon attenuation, scatter and partial volume would collectively improve the detection and estimation task [15]. This is particularly important for small structures and in small energy radionuclides such as I-125 [16].

Unlike PET, single photon emitting radiopharmaceuticals have several features in the context of molecular imaging such as cost and wide availability of the radioligands as well as relative ease of labeling. Small animal imaging using preclinical scanners and PET radiopharmaceuticals showed better capability in tracer kinetic studies when compared to its SPECT counterparts. PET compounds have been extensively used in compartmental modeling and kinetic analysis. Furthermore, small animal PET scanners showed a large axial field of view such that distant tissues/organs can be covered when image derived input function such as left ventricle is sought for calculations.

### 2.3. Pinhole Geometry: Pros and Cons

Hal Anger used a pinhole collimation which is an important element when we come across *μ*SPECT imaging. The early work done on small animal imaging using SPECT tracer was to use a gamma camera equipped with pinhole collimator(s) of very small aperture size. Although parallel hole is the most commonly used collimator in many nuclear medicine procedures, pinhole imaging has a well-recognized role particularly for small organs such as thyroid and parathyroid imaging. In bone joints as well as in some pediatric studies, pinhole can also improve the spatial resolution by magnifying small structures of different tracer uptakes.

In recent years, pinhole geometry was found an increasing interest in designing SPECT scanners with superb spatial resolution and this has been attained by minimizing the aperture size to sub-millimeter range and specialized collimator geometry. However, the cost paid for this improved spatial resolution is a reduction of the detection efficiency. The later was partially tackled by increasing the number of holes for improvement of count collection and statistical quality. Pinhole geometry is not similar to parallel hole geometry where one-to-one magnification is achieved. The geometric magnification provided by pinhole geometry is a function of the object distance from the aperture as well as distance of the aperture from the detector surface in addition to the effective aperture diameter. Nothing is free, this takes place with a reduction of the imaging field of view. Another problem encountered when pinhole collimator is used in tomographic acquisition is data insufficiency and the resulting images could suffer from reconstruction errors.

Image reconstruction using iterative techniques have solved many problems that were not possible to achieve with analytic approaches. In clinical and preclinical arena involving both SPECT and PET imaging, iterative reconstructions were found superior to analytic approaches in many aspects of diagnostic quality and quantitative accuracy. *μ*SPECT imaging has received large benefit from the use of iterative reconstruction by incorporating as many degrading factors in the system matrix. Besides its treatment to image noise, iterative reconstruction for pinhole geometry can correct for photon attenuation, scatter, and system response function. Edge penetration and parallax errors can also be modeled in the reconstruction scheme reducing the blurring effect allowing for enhanced spatial resolution [17].

### 2.4. Detectors

A conventional gamma camera can be used by manufacturing pinhole collimators of very small aperture size. It provides large field of view such that better magnification can be achieved. The other alternative is to use pixilated detectors that have better intrinsic properties or semiconductor detectors that fit with the resolution requirements of small animal imaging and, meanwhile, better than that provided by the conventional gamma camera. Regardless of their cost, semiconductor detectors are more compact and allow for system portability and can be manufactured in pixilated structure providing better spatial resolution.

Using a large field of view gamma camera serves to improve the magnification by providing large projection area onto the detector surface for the subject under investigation. However, some recent scanners are implementing pixilated detectors that have an intrinsic resolution equivalent to the segmentation size. This, to some extent, obviates the need to use detector width of size equivalent to the standard clinical gamma camera [5]. Thallium-activated sodium iodide NaI(Tl) crystal is the conventional scintillator used in most clinical designs. However, there are also some scintillators that have been used such as Cesium Iodide-Thallium doped and Cesium Iodide-Sodium doped and Yttrium Aluminum Perovskite (CsI(Tl), CsI(Na), and YAP, resp.). New designs of photodetectors such as position sensitive PMT, avalanche photodiode and position sensitive avalanche photodiode can be of value in *μ*SPECT systems. For example, Funk et al. [18] have designed a multipinhole small animal imaging based on position sensitive avalanche photodiode (PSAPD) detectors coupled to CsI(Tl) scintillator. The system showed submillimeter spatial resolution and high detection efficiency when compared to dual head gamma camera, permitting shortened acquisition time and a reduced injected dose.

### 2.5. Designs

Several designs were proposed for pinhole geometry, including rotating gamma camera, stationary detector but rotating collimators, or completely stationary camera [19]. In U-SPECT II (MILabs, The Netherlands), the three-headed gamma cameras is equipped with interchangeable multihole collimators that can achieve high spatial resolution [20]. The collimator is cylindrical in shape with relatively large number of pinholes (i.e., 75), providing a good count collection for the high spatial resolution. A resolution of 0.35 mm can be achieved with an aperture size of 0.35 mm while a spatial resolution of 0.45 mm can be obtained with a 0.6 mm gold pinhole aperture size. The values are less in case of rat imaging (0.8 mm) using the standard whole body rat collimator. A recent release of the MILab company is the simultaneous acquisition of SPECT and PET tracers with resolution that can reach below 1 mm for the former [21].

The Inveon is another commercial design provided by Siemens Medical Solutions. The scanner is a trimodality imaging system that has three imaging modules namely PET, SPECT and CT. The SPECT and CT are coplanar and mounted on the same rotating gantry. The SPECT portion can be two or four 150 mm × 150 mm NaI(Tl) pixilated detectors (2.2 mm pitch) and 10 mm crystal thickness. The heads can be equipped with various parallel-hole, single- or multipinhole collimators, including mouse general body as well as mouse brain imaging with possible submillimeter spatial resolution. 

 Another two commercial *μ*SPECT designs provided by Gamma-Medica Ideas and Bioscan. The Triumph Trimodality scanner (Gamma Medica, Inc) is an integrated SPECT/PET/CT hardware and software platform designed for small animals in preclinical and biomedical research applications. The system combines PET (LabPET), SPECT (X-SPECT) and CT (X-O) modalities. The SPECT module utilizes solid-state cadmium zinc telluride (CZT) detector technology. It provides opportunities to scan individual organs or whole body images. The SPECT system accommodates single and multiple pinhole collimators as well as parallel hole collimators to address a broad range of study needs. It can be configured to have 1,2,3 or 4 CZT cameras providing a variety of spatial resolution, detection sensitivity, and scanning field of view [20]. 

**Figure 2:**
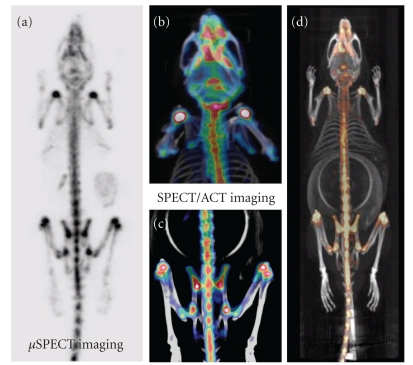
Invivo SPECT and SPECT/CT with ^99m^Tc-MDP in C57BL/6 mice (3 hrs after injection dose 120 MBq i.v.). Note the high uptake in glenohumeral, the hip, and the femorotibial joints as shown in the whole body mouse (a). In (b) and (c), upper and lower extremities are shown where SPECT and CT images are coregistered. The whole body fused SPECT and CT is shown in (d). Images were acquired with the Inveon system (Siemens Medical Solutions) using dual detectors each mounted with a 5-pinhole collimator. The pinhole aperture size was 1.0 mm.

The Bioscan system has a four-detector head that consist of NaI(Tl) crystal. The scanner uses the spiral path to scan the object (24 to 270 mm) and also has stationary and circular detector motion. It has a variety of collimator options that can reach <1 mm spatial resolution in addition to high detection sensitivity [22]. It uses a patented multiplexed-multipinhole collimator design that can reach 36 pinholes or eve more. Commercially available *μ*SPECTs are shown in Figure 2.

### 2.6. Hybrid SPECT Systems

**Figure 3:**
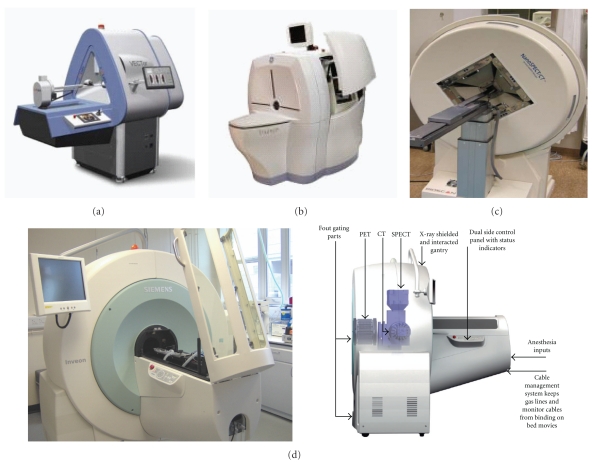
Some commercial preclinical SPECT systems. (a) A preclinical hybrid PET/SPECT device, the VECTor (Image courtesy of MI Labs), (b) Triumph Trimodality scanner (courtesy of Gamma Medica), (c) NanoSPECT (courtesy of BioScan, Inc), and (d) Inveon system (courtesy of Siemens Medical Solution) with internal components demonstrated (courtesy of Siemens Medical Solution).

*μ*SPECT scanners can produce functional images with high spatial resolution; however, anatomical correlation using structural imaging modalities is still needed. For this reason, CT or MRI have been incorporated in some *μ*SPECT systems. In addition to SPECT/CT and SPECT/MRI other hybrid systems such as SPECT-optical devices have also been investigated [23, 24]. The common underlying idea is to get and extract more information about the biological question in one imaging session and preferably with the same spatial and/or temporal framework.  Figure 3 shows bone imaging in mouse using an SPECT/CT preclinical scanner. CT devices provide several advantages to the SPECT. They produce high resolution anatomical images in addition to generating a subject-specific attenuation map able to correct for photon attenuation. MRI machines can have a better soft tissue contrast, not relying on ionizing radiation, and provide high spatial resolution as mentioned earlier.

microCT (*μ*CT) has been advanced in the last few years providing a spatial resolution in the order of few microns. A resolution of 10 *μ*m or even better can be achieved giving more insights into structural abnormalities for *in vivo* as well as *ex vivo* samples. Nowadays, *μ*CT is not only for attenuation and anatomical localizations but the benefits were extended to blood vessels imaging which is known as CT angiography. A number of reports were recently released discussing the utility of *μ*CT in many preclinical applications [25, 26]. SPECT/MRI systems were also designed, and it is worthy mentioning that image of the year 2008 (in the annual meeting of the Society of Nuclear Medicine) was selected where diabetic feet using SPECT were coregistered with the patient MRI providing anatomolecular diagnosis of the extent and location of the disease. In preclinical context, the interest was given to PET/MRI rather than SPECT/MRI. However, new photodiodes that are less prone to magnetic fields can be very helpful in such designs.

## 3. Radiopharmaceuticals

Molecular imaging is an emerging field of study that deals with imaging of disease on a cellular or genetic level rather than on a gross level [27]. With the emergence of the new field of molecular imaging, there is an increasing demand for developing sensitive and specific novel imaging agents that can rapidly be translated from small animal models into patients. SPECT and PET imaging techniques have the ability to detect and serially monitor a variety of biological and pathophysiological processes, usually with tracer quantities of radiolabeled peptides, drugs, and other molecules at doses free of pharmacologic side effects [28].

### 3.1. Radiolabeled Molecular Imaging Probes (RMIPs)

RIMPs are highly specific radiolabeled imaging agents used to visualize, characterize, and measure biological processes in living systems. Both, endogenous molecules and exogenous probes can be molecular imaging agents. The ultimate goal of molecular medicine is to treat the disease in its early stages with an appropriate patient-specific “targeted molecular therapy.” In order to achieve this goal, it is essential to develop highly specific RMIPs. In the design and development of an ideal RMIP, it is important to identify first a molecular imaging probe (MIP), which may be a biochemical or a synthetic molecule, specific for a biological process (such as metabolism, angiogenesis, and apoptosis) or a molecular target (such as hexokinase, thymidine kinase, and neuroreceptor) in an organ, or tissue of interest.

**General Rules for the Design of RMIPs**

An ideal RMIP should be designed to fulfill the following characteristics [29].

- Rapid plasma clearance to reduce blood pool background in the target tissue.
- Rapid washout or clearance from non specific areas.
- Low nonspecific binding and preferably no peripheral metabolism.
- High membrane permeability and intracellular trapping.
- Target specificity and high affinity for molecular targets.
- Specific activity must be high to prevent saturation of specific binding sites.
- Tissue distribution, localization, and target binding should be favorable for developing simple kinetic modeling to estimate quantitative data.
- Radiation dosimetry of RMIP must be favorable for multiple diagnostic imaging studies (if necessary).
- Synthesis of RMIP must be rapid and suitable for automation using automated synthesis modules.

**Radiolabeling of RMIPs**

Generally, the radiolabeling process of molecular imaging agents can be categorized as follows.

**Isotope Exchange**

Where the preparation is obtained by direct exchange of stable atom(s) of an element in a molecule with one or more nuclide of a radioisotope of the same element.

**Introduction of Foreign Element**

This is the most common method of radiolabeling of RMIPs, however, the RMIP will have a chemical structure and in vivo behavior different from that of the parent MIP.

**Metal Chelation**

In this method, a chelating agent (radiometal such as ^99m^Tc and ^111^In) is being introduced into an organic compound producing a ligand with different biological and chemical features than both the conjugated two partners. Certain peptides and monoclonal antibodies can successfully be labeled by the metal chelation procedure but only in the presence of a bifunctional chelate (BFC) by conjugation with the peptide or protein first and then bind the radiometal to the BFC conjugated molecule.

**Classification of RMIPs**

Based on their clinical utility and the nature of application for which they are designed as tools in the drug development program, four classes of RMIPs have been identified [30].

- *Radiolabeled drug substance *in which the cold stable atom is replaced by a radioisotope of the same element, which can be used for assessing the pharmacokinetics and biodistribution of the parent drug.
- *Radioligand *with good binding affinity for a biological target, which can be used to evaluate the effect of other unlabeled compounds at that target.
- *Pathway marker *interacting with one component of a set of related biological molecules, which may be used to probe the overall status of that system.
- *Biomarker*, or surrogate marker, which provides a more general readout at the level of cell or organ for a specific biological process.

#### 3.1.1. Peptides and Proteins

**(1) Radiolabeled Monoclonal Antibodies (MAbs)**

Antibodies are immunoglobulins (Ig) produced *in vivo* in response to the administration of an antigen to a human or animal tissue and bind specifically to this antigen forming an antigen-antibody complex [31]. Since the advent of hybridoma technology for production of MoAbs in 1975 [32] which was designed originally as an in vivo tumor localizing agents, only few have reached a point of proven clinical utility [33].Labeling of MAbs can be accomplished with several radionuclides, among which In-111, Tc-99m, I-131, I-123, and I-125, where most of them are commonly used in nuclear medicine [34] and listed in Table 1. A number of monoclonal labeled antibodies using In-111 or Y-90 radionuclides are described in Table 2.

**(2) ^99m^Tc-labeled Monoclonal Antibodies**

**Arcitumomab (CEA Scan)**

Carcinoembryonic Antigen (CEA) or CEA-Scan kit (introduced by Mallinckrodt Medical) as a single dosage kit contains the active ingredient Fab^−^ fragment of arcitumomab, a murine monoclonal antibody IMMU-4. CEA is expressed in a variety of carcinomas, particularly of the gastrointestinal tract (GIT) and can be detected in the serum. IMMU-4 is specific for the classical 200 kDa CEA that is found predominantly on the cell membrane. ^99m^Tc-CEA-Scan complexes the circulating CEA and binds to CES on the cell surface. Fab^−^ fragment of arcitumomab is cleared rapidly by urinary tract and plasma clearance due to its small particular size [38]. The IMMU-4 antibody is targeted against the carcinoembryonic antigens of the colorectal tumors, and, therefore, ^99m^Tc-CEA-scan is used for the detection of recurrence and/or metastatic carcinomas of the colon or rectum particularly when high levels of CEA are detected [39]. However, it is an uncommon procedure following PET/CT scan.

**Sulesomab (LeukoScan)**

The kit vial contains the active ingredient Fab^−^ fragment, called sulesomab, obtained from the murine monoclonal antigranulocyte antibody, IMMU-MN3. It is a single-dose kit introduced by Immunomedics Europe in 1997. The labeling yield should be more than 90% [40]. ^99m^Tc-sulesomab targets the granulocytes, and therefore is primarily used to detect infection and inflammation, particularly in patients with osteomyelitis, joint infection involving implants, inflammatory bowel disease, and diabetic patients with foot ulcers [41].

**Annexin V (Apomate)**

Annexin V is a human protein with a molecular weight of 36 kDa has a high affinity for cell membranes with bound phosphatidyl serine (PS) [42]. In vitro assays have been developed that use Annexin V to detect apoptosis in hematopoietic cells, neurons, fibroblasts, endothelial cells, smooth muscle cells, carcinomas, and lymphomas. ^99m^Tc-annexin V has also been suggested as an imaging agent to detect thrombi in vivo, because activated platelets express large amounts of PS on their surfaces [43].

**(3) Radiolabeled Peptides**

High background activity difficulties which usually appear when imaging with radiolabeled MAbs which is attributed to the slow tumor uptake and plasma clearance due to their relatively large molecular sizes. This can be mitigated by peptides whose molecular size is smaller than that of proteins and where the peptidases can act for rapid excretion by degradation of the peptides. Peptides have been labeled with ^111^In and ^99m^Tc in the same manner of the monoclonal antibodies. Some useful labeled compounds are listed in Table 3.

#### 3.1.2. RMIPs for Metabolism

Metabolic imaging can be achieved using natural or exogenous radiolabeled substrates which participate in the particular metabolic process. The design of such tracers is based on the physiological concepts such as turnover of oxygen, glucose, amino acids, fatty acids, or DNA precursors. Commonly, ^123^I and ^99m^Tc derivatives are used as SPECT tracers for this function, however, the obvious chemical changes occur with this conjugation which can alter the physiological properties of the tracer module limits their application in molecular imaging.

**(1) Glucose Metabolism**

Although there are intensive research trials to find a sugar derivative labeled with ^123^I and ^99m^Tc, none of these were able to provide an SPECT substitute for ^18^F-FDG to act as glucose metabolism imaging agent [51–53].

**(2) Amino Acid Metabolism**

Diagnosis of various neurological diseases as well as tumor evaluation (detection, grading and therapy monitoring) can be obtained by quantitative assessment of protein synthesis rate that is provided when using radiolabeled amino acids as a radiolabeled molecular imaging probe (RMIP). Radiolabeled amino acids pass the blood-brain barrier and are accumulated in tissues via a specific amino acid transport system [54]. _ L_-3-^123^I-Iodo-*α*-methyltyrosine (^123^I-IMT) is a good example of radiolabeled amino acids, it is evidenced that it accumulates in the brain via a specific facilitating L-amino acid transport system. In analogy with PET, IMT is not incorporated into proteins [55] and its uptake reflects amino acid transport [56]. IMT can be prepared by electrophilic substitution via in situ oxidation of ^123^I-Iodide by chloramines-T, hydrogen peroxide, iodogen, or iodate with radiochemical yields of 70%–80%.

**(3) Nucleosides Metabolism (Gene Reporter Imaging)**

Nucleosides or nucleoside analogs can be transported across the cell membrane by selective transporters and can then be phosphorylated intracellularly by specific kinases to the corresponding phosphate derivatives, and, ultimately, they can be incorporated into DNA. As thymidine kinase 1 (tk1) shows an S-phase dependant expression, the intracellular accumulation of labeled nucleosides that are substrates for TK1 reflects DNA synthesis and thus tumor proliferation. I-131-[57] and I-124-[58] labeled 2-arabino-fluro-5-iodo-2-deoxyuridine (FIAU) has been used successfully as nucleoside analog for tumor proliferation detection. Nucleoside derivatives that are selective substrates for herpes simplex virus thymidine kinase (HSVtk) have been developed for the in vivo visualization of transgene expression using the HSVtk gene as a reporter gene. Radioiodine labeled uracil compounds (e.g., FIAU) are widely applicable derivatives.

**(4) Hypoxia Imaging**

Hypoxia, a condition of insufficient O_2_ to support metabolism, occurs when the vascular supply is interrupted, as in stroke or myocardial infarction, or when a tumor outgrows its vascular supply. When otherwise healthy tissues lose their O_2_ supply acutely, the cells usually die, whereas when cells gradually become hypoxic, they adapt by upregulating the production of numerous proteins that promote their survival. These proteins slow the rate of growth, switch the mitochondria to glycolysis, stimulate growth of new vasculature, inhibit apoptosis, and promote metastatic spread [59].

Most hypoxia markers contain a nitroimidazole moiety as a reactive chemical species. Nitroimidazoles can be used as probes to detect hypoxia as they are reduced intracellularly in all cells, but in absence of adequate supply of O_2_, they undergo further reduction to more reactive products which bind to cell components and are finally trapped in the hypoxic tissue [60]. ^99m^Tc-O-propylene-amine-oxime (^99m^Tc-pano or BMS-181321) was validated as a proper hypoxia imaging agent in hypoxic myocardium, acutely ischemic brain and solid tumors [61]. BMS-194796 and ^99m^Tc-HL91 have also been designed as RMIP for imaging hypoxia, however, none of these three probes has been commercially available. Iodine-123 labeled iodoazomycin arabinoside (IAZA) has been validated in animal model in the preclinical phase [62], but no clinical studies with this agent have been reported so far.

**(5) Cell Labeling**

The most common applications of In-111 are in labeling blood cells (white blood cells (WBC) and platelets) for imaging inflammatory processes and thrombi [63]. In blood cell labeling, the plasma transferrin competes for the In-111 and reduces the labeling efficiency because In-111 binds with higher efficiency to transferrin than blood cells, and, therefore, isolation of the desired blood component from plasma permits easy labeling of either platelets or WBCs. ^99m^Tc-HMPAO is primarily used in brain perfusion imaging, although it is used for leukocyte labeling substituting ^111^In-Oxine. Stabilization of the ^99m^Tc-HMPAO primary complex is required due to the high degradation rate of its radiochemical purity. This could be achieved by adding stabilizers like methylene blue in phosphate buffer or cobalt (II)-chloride to the reaction vials [64], however, these reagents should not be used when the complex formulation is designed for labeling of leukocytes.

**(6) Iodine-123 as an RMIP**

I-123 is the preferred thyroid imaging agent imparting 1% of thyroid dose per microcurie when compared with I-131. ^123^I-labeled compounds are commonly used as ^123^I-MIBG for adrenal scan, ^123^I-OIH for tubular renal scan, and ^123^I-iodoamphetamine (^123^I- IMP) for cerebral perfusion scan [28].

*^123^I-Ioflupane (DaTScan)* is a widely used ^123^I derivative for detection of the loss of nerve cells in an area of the brain called the striatum which release dopamine, a chemical messenger, and therefore, it will be useful in distinguishing between Parkinson's disease and essential tremor (tremors of unknown cause) with a sensitivity of 96.5% [65]. It is also used to help distinguish between “dementia with lewy bodies” and Alzheimer's disease with 75.0% to 80.25% sensitivity [66].

Dopamine transporter (DAT) imaging with tropane derivatives such as FP-CIT (^123^I-Ioflupane) and *β*-CIT has been developed to directly measure degeneration of dopamine presynaptic terminal and may be used to quantify changes in DAT density. Ioflupane binds specifically to structures of the nerve cells ending in the striatum area of the brain that are responsible for the transportation of dopamine. This binding can be detected using tomographic imaging [67].

## 4. SPECT in Preclinical Applications

The application for imaging modalities in preclinical models is highly valuable as it has a great scope for noninvasively studying dynamic biological processes at the molecular and cellular level. The noninvasive nature of imaging provides advantages in investigating the onset and the progression of disease, assessing the biological effects of drug candidates and assisting in the development of disease biomarkers and monitoring the therapeutic effectiveness of new treatment and/or pharmaceuticals. This technology plays a key role in bridging bench studies of disease modelled in vitro to their implementation in clinically relevant animal models of diagnostic or therapeutics for their translation into the clinics. In fact, the implementation of imaging in rodents has a great relevance because of the widespread use of genetically modified mice in biomedical research and the need to characterise the *in vivo* anatomical and functional phenotypes of animal disease models. Another advantage of imaging modalities developed for small animals is that the technology can relatively be translated directly for application to clinical practice.

### 4.1. Cardiovascular Imaging

Preclinical SPECT systems have a great scope of applications in cardiovascular research, including the study of myocardial functions (e.g., ejection fraction, regional wall motion abnormalities, perfusion, tissue viability, oxygen consumption, and glucose metabolism [68]) and the investigation of several vascular disorders, including coronary artery disease and related disorders, such as ischemia, infarction and atherosclerosis [69]. Moreover, *μ*SPECT has great applications for developing and testing diagnostic tracers which could assist in understanding the prognosis of disorders and assess new therapeutic approaches for cardiovascular lesions.

^99m^Tc-labelled radiopharmaceuticals for SPECT imaging have been applied to demonstrate tissue viability and perfusion status in animal models of ischemia and/or impaired myocardial perfusion [70, 71]. Cardiac and respiratory motion is one of the major challenges when imaging rodent (mouse heart rate: 400–800 beats/min). Gated acquisition is therefore required to minimise any movement artefacts. Indeed, ECG-gated micro SPECT can yield accurate measurements of left ventricle volumes and ejection fraction in rats and mice [72, 73]. 

Another key area is the visualization of necrotic tissues and related tracers during myocardial infarction (MI). Some studies have assessed myocardial ischemia in rat heart models after left coronary artery occlusion by using ^99m^Tc-glucarate [74]. In vivo visualization of necrosis may help to detect MI at early stages and may provide a good approach for evaluating the antinecrotic effect of developing drugs for ischemic heart disease. On the same vein, the visualization of apoptotic cell death is another important target for non-invasive imaging [75]. Hence, the development of tracers (e.g.,^99m^Tc-Annexin) that bind to apoptotic cells is a very useful tool for in vivo analysis, especially to investigate apoptotic cell death in cardiomyocytes and the efficacy of cell-based therapies. 

Angiogenesis imaging is a key protective/remodelling mechanism in myocardial infarction. Imaging such a mechanism is important for the understanding of infarct healing and post-MI remodelling. Vascular growth factors such as *α*_v_ *β*_3_ integrins have been used as targeted tracers to investigate angiogenesis in postinfarct animal models, with In-111-labelled *α*_v_ *β*_3_ targeted radiotracers in hypoperfused myocardial regions [76].

Another relevant area of cardiovascular imaging is the development of methods to characterise the formation and prognosis of atherosclerotic plaques. Plaque rupture results in severe cardiac events including MI and sudden death, hence, there is an important need for developing tools that can assist in predicting the plaques vulnerability to rupture. Not all the plaques carry the same risk, and the criteria for imaging their vulnerability relies on the detection of inflammatory cell infiltration, platelet aggregation, tissue matrix degradation, large lipid contents and apoptosis. Radiolabeled Annexin and Z2D3 targeted to apoptotic macrophages and smooth muscle cells, respectively, have been used as SPECT tracers to study the pathophysiology of atherosclerosis in animal models [77, 78]. One of the main challenges for imaging plaques is their anatomical localization, as high spatial resolution is needed to image such a small anatomical structure in a motile vessel, hence the importance of co-registering SPECT acquisition with other imaging modalities such as micro-CT.

### 4.2. Imaging Stem Cells

With advances in research of stem cell-based therapies, the application of imaging technologies may be useful to validate their efficacy and safety in preclinical models, in particular, for studying tracking and engraftment of transplanted cells, assessing their viability, function and differentiation status in addition to monitoring their ability to promote regeneration [79]. Stem cells can be labelled with radionuclides before transplantation. For example, stem cells labelled with the SPECT radiolabels ^111^In-oxyquinoline have been successfully imaged after transplantation in rat and porcine models of myocardial infarction [80, 81], but, because of the short half-life of the radionuclide (e.g., ^99m^Tc: 6.02 h; ^111^In: 2.8 days) and because the activity may still be present after transplantation even if the cell have died, this method may only be applicable for a short-term cell tracking and assessing stem cell homing after transplantation.

To investigate not only long-term engraftment of stem cells but also their viability, a gene imaging approach may be more appropriate. In this case, gene expression is assessed by reporter genes constructs which are translated into a protein and interact with an exogenously given probe (radiolabeled for SPECT detection), resulting in a signal that can be monitored non-invasively. Reporter genes are incorporated into the cells before transplantation, and if the cell remains alive after engraftment, the protein, which is the main target for the nuclides, will be encoded (e.g., enzyme, cell surface receptor). Conversely, the reporter gene will not be expressed if the cell is dead [82].

As mentioned earlier, one of the reporter genes mostly used for SPECT imaging is based on the production of an intracellular enzyme (e.g., herpes simplex virus type 1 thymidine kinase [HSV1tk]) that phosphorylates an exogenously administered substrate that is retained in the cell because of its negative charge. Although normal mammalian cells (without the HSV1-tk) do carry the enzyme, it only minimally phosphorylates the radionuclide probes used in this system. Conversely, in cells carrying the HSV1-tk, the exogenously administered probe undergoes significant phosphorylation and intracellular retention, leading to a robust signal-to-background ratio and enabling accurate monitoring of these cells [83]. This imaging approach has been recently used to monitor the distribution of transplanted human embryonic stem cell derivatives in a live mouse model over a long period of time, up to 3 months [84].

Another approach consists of the encoding of the sodium-iodide symporter (NIS), a thyroid transmembrane protein that, under physiologic conditions, transports iodine into the cells in exchange for sodium. It has the advantage that it can be used for PET (with ^124^I as the tracer) and SPECT imaging (using ^123^I or ^99m^Tc-pertechnetate as tracer). This approach has been used to monitor activity of cardiac-derived stem cells after transplantation to a rat infarct model, confirming the visualization of cells up to 6 days after transplantation [85]. With rapid advances in stem cell research, and with high demands for testing their regenerative potential in preclinical model, noninvasive stem cell imaging will play a critical role, and we can foresee more studies requiring long-term monitoring of stem cells in preclinical models of disease.

### 4.3. Oncologic Applications

Imaging techniques play a potential role in preclinical cancer research, enabling sequential analysis of deep-seated tumors and metastases including studies of basic biological processes, tissue pharmacokinetics and pharmacodynamics responses to treatments. Imaging of cancer cells targets have different biological procedures including overexpression of receptor, activated enzymes or relocated molecules, apoptotic levels, sustained angiogenesis, unlimited replicative potential and invasion of tissue, and metastasis [86].

Imaging gene expression *in vivo* is very relevant in cancer preclinical models as it allows the characterization of dynamic changes in several deregulated pathways in cancer cells. As previously mentioned, HSVtk genes are typically used for SPECT, enabling noninvasive imaging of tumor cell growth as demonstrated in an experimental mouse model for lung metastases expressing after injection of HSV1-tk cells [87]. This model may be proven very useful for assessment of anticancer and antimetastases therapies in preclinical efficacy models.

Another used approach is the imaging receptors that are overexpressed in cancer cells and can be used for prognosis and for following therapeutic targeting. This has been successfully applied preclinically as well as clinically by targeting prostate-specific membrane antigen (PSMA). This receptor is overexpressed on the cell surface of prostate cancer cells and provides a useful target for prostate tumor imaging and therapy. As mentioned previously, radiolabeled monoclonal antibodies, such as ^111^In-Capromab pendetide (ProstaScint), are currently available to detect prostate cancer but suffer from problems associated with poor delivery because of their large size [88]. The feasibility of imaging PSMA receptor expression with low-molecular-weight, high-affinity PSMA ligands labeled with [^125^I] NaI/Iodogen for SPECT was demonstrated in a study using a prostate tumor mouse model [89].

SPECT technology is extensively used as a diagnostic tool for bone metastases in the clinic. Bone scintigraphy with ^99m^Tc-labelled diphosphonate is a widely used method for the detection of bone metastases, and other bone disorders. This technique provides a high sensitivity and is able to survey the whole skeleton but unfortunately does not provide enough anatomical resolution to allow precise localization of the radiotracer high uptake lesion. The implementation of a SPECT/CT multimodality system can partly overcome this disadvantage, allowing a coregistration of the functional and the anatomical imaging component, resulting in precise anatomical localisation of the radiotracer. Studies have reported the use of Tc-99m-labeled diphosphonates compounds (e.g., methylene-diphosphonate (MDP)) to detect metastatic bone lesions in immunocompromised mouse models injected with cancer cells. Overall, the application of SPECT imaging in cancer research while remaining challenging have already had a remarkable impact providing new insights into the dynamics of cancer growth, invasion, and metastases, being possible to visualize gene expression, molecular pathways and functional parameters in preclinical models of cancer.

### 4.4. Neuroimaging Applications

The use of SPECT in preclinical functional neuroimaging provides an excellent application for understanding the pathophysiology of central nervous system (CNS) disorders, including the mechanisms of neurodegeneration, neuropharmacology related to drug abuse, and testing therapeutic strategies. As mentioned earlier, one of the strength of SPECT over other functional modalities such as the PET is the ability to get a spatial resolution below 1 mm, allowing detailed structural and functional information of different region of the brain in animal models. Also, SPECT radioligands have relative longer half-lives, which permits prolonged dynamic function studies and provides a simultaneous dual tracer imaging. The use of pinhole SPECT facilitates accurate and quantitative imaging. Indeed, specific radioligands have been used to study the dopaminergic, serotonergic, and cholinergic neurotransmission system in vivo [90].

 SPECT has been applied to study basic mechanisms of degeneration in Parkinson's (PD) and Alzheimer's disease (AD). PD is characterized by a progressive loss of dopaminergic neurons. Animal models of neurodegeneration have been used to evaluate novel radioligands and to study their binding in the dopaminergic synapsis. In vivo quantification of the presynaptic dopamine transporter (DAT) activity which regulates the synaptic dopamine is feasible in the rat striatum using the ^ 123^I-*N*-*ω*-fluoropropyl-2*β*-carbomethoxy-3*β*-(4-iodophenyl)-nortropane (^123^I-FP-CIT) as a DAT radioligand [91], as outlined earlier. This technology is of particular interest for investigating the interrelation of synaptic dopamine and DAT in animal models of PD. Similarly, Acton et al. have characterized the occupancy of dopamine receptors in the mouse brain [92]. SPECT imaging has improved the early diagnosis of AD in patients by detecting the onset of progressive neurodegenerative disorders and vascular brain pathology causing dementia. 

The thioflavin derivative, 6-iodo-2-(4′-dimethylamino-) phenyl-imidazo [1,2-a] pyridine, IMPY, which is readily radiolabeled with ^125^I/^123^I [^123^I] IMPY was assessed *ex vivo* in a transgenic mouse model of AD by labeling the deposition of amyloid plaque that is linked to the pathogenesis of AD [93], showing a good binding with brain tissue homogenates of confirmed Alzheimer's disease patients. Overall, these preclinical studies support the use of SPECT for functional imaging in preclinical models of CNS disorders, facilitating the efficacy of translational studies into this field with a direct relevance for developing clinical diagnostic tools and efficacious therapies.

### 4.5. Drug Discovery

Preclinical imaging has a key role in the drug development, in particular for validating drug targeting, safety, and efficacy. One of the main applications is the validation in drug binding assays to specific targeted areas, by directly labeling a drug to determine its distribution and pharmacokinetics. This approach is very useful for validating delivery roots and the specificity of novel therapeutic drugs or imaging agents. SPECT imaging has been applied to measure the binding potential of targeted nuclide [^123^I] Iodobenzamide to the dopamine transporters in the rat brain after specific treatments [94]. Similarly, ^99m^Tc-labeled liposomes migration has been studied after intratumoral administration to tumor xenograft models in nude rats [95]. Specific skeletal targeted probes (125I labeled) were investigated with particular emphasis on the pharmacokinetics and biodistribution following intravenous administration. This has proved to be of great potential for validating the efficacy of animal models of osteoporosis and other skeletal diseases [96].

Imaging technologies are also very useful for safety validation, as they can measure the functional response of organs to a tested drug candidate, providing information on any toxic or secondary effects related to the treatment. Radiopharmaceuticals that bind to apoptotic cells (e.g., ^99m^Tc-Annexin) have been used preclinically to validate the possible toxicity effects in developing therapies [97]. Other examples on secondary response to the administration of a candidate drug in preclinical models include measurements in blood flow changes [98] and infiltration of inflammatory cells [99].

Another important application is the development and validation of imaging biomarkers, a surrogate imaging product that simulates a biological compound and/or has some biologic link to the disease process. These biomarkers are becoming very useful to assess therapeutic actions of pharmaceuticals, providing a non-invasive imaging tool for validating the efficacy of treatment. Advances in proteomics and genomics are leading to the discovery of new biomarkers, promoting the use of functional imaging for their validation and translation into the clinics [100].

## 5. Conclusion

SPECT imaging has a well-defined role in the world of molecular imaging. Recent advances in dedicated preclinical systems are able to provide high spatial and temporal resolution as well as high detection efficiency with more potentiall for further improvement. Hybrid SPECT imaging systems would serve characterizing biological phenomena in one imaging session. Reliable animal models that, mimic human diseases is another innovative field that when combined with SPECT technology will reveal more insights into early disease detection, development of new tracers/therapeutics and treatment strategy. SPECT imaging has a large potential in molecular medicine, and many novel approaches are expected in the near future.

## Figures and Tables

**Table 1:** Commonly used single photon emitting radionuclides.

Radionuclide	Half-life	Energy	Mode of decay
Tc-99m	6.02 h	142 keV	IT (100%)
I-131	8.03 days	364 keV	*β* ^−^ (100%)
I-123	13.22 h	159 keV	EC (100)
In-111	2.80 days	171, 245 keV	EC (100%)

IT: isomeric transition, *β* ^−^: beta-minus, EC: electron capture.

**Table 2:** List of In-111- and Y-90-labeled monoclonal antibodies.

Compound	Description	Applications	Remarks
In111-Capromab pendetide (ProstaScint)	A conjugation between the murine antibody 7E11.C5.3 and ^111^In in the source of ^111^InCl_3_ by the action of the GYK-DTPA as a chelating agent.	Indicated for use in immunoscintigraphy, proven prostate carcinoma and patients who have undergone a prostatectomy and have rising prostate specific antigen (PSA) values and equivocal nonevidenced metastasis.	It is not indicated with patients with a high clinical suspicion of occult metastatic disease or for screening of prostate carcinoma.

In111-Satumomab pendetide (OncoScint)	It contains the murine MAb B72.3 which is directed to tumor-associated glycoprotein. It is labeled with ^111^InCl_3_ by conjugation with the chelating agent, GYK-DTPA.HCl.	Used for the detection of colorectal and ovarian cancers [35].	After an incubation time of 30 min, the labeled mixture is suitable for use in the first 8 hours.

In111-Imciromab pentetate (MyoScint)	An antibody produced against myosin in the cell culture, and therefore binds to the heavy chain of myosin after in vivo administration.	Detection of myocardial infarction.	Contains the Fab fragment of a murine monoclonal antibody that is covalently bound to DTPA giving ^111^In -Imciromab pentetate.

In-111 and Y90-ibritumomab tiuxetan (Zevalin)	*Zevalin* consists of a murine monoclonal anti-CD20 antibody covalently conjugated to the metal chelator DTPA, which forms a stable complex with ^111^In for imaging and with ^90^Y for therapy.	^90^Y-ibritumomab tiuxetan is used for the treatment of some forms of B cell non-Hodgkin's lymphoma, a myeloproliferative disorder of the lymphatic system while its ^111^In derivative is used to scan the predicted distribution of a therapeutic dosage of ^90^Y-ibritumomab in the body [36].	The antibody binds to the CD20 antigen found on the surface of normal and malignant B cells (but not B cell precursors) allowing radiation from the attached isotope (Yetrium-90) and the cytotoxicity induced by the antibody serve to eliminate B cells from the body allowing a new population of healthy B cells to develop from lymphoid stem cells [37].

Rituximab	An earlier version of anti-CD20 antibody and has also been approved under the brand name Rituxan for the treatment of non-Hodgkin's lymphoma (NHL).	It was approved for the treatment of patients with relapsed or refractory, lowgrade or follicular Bcell NHL, including patients with rituximab refractory follicular NHL.	In September 2009, ibritumomab received approval from the FDA for an expanded label for the treatment of patients with previously untreated follicular NHL, who achieve a partial or complete response to first-line chemotherapy.

**Table 3:** Tc99m- and In-111-labeled peptides.

Compound	Description	Uses	Remarks
In-111 labeled compounds			

In-111 pentetreotide (OctreoScan)	^111^In has been conjugated to octreotide as DTPA chelated compound to form the labeled somatostatin tracer (^111^In-DTPA-octeriotide).	Octreoscan is an agent designed for the scintigraphic localization of primary and metastatic somatostatin receptor positive neuroendocrine tumors, such as carcinoids, gastrinoma, neuroblastomas, pituitary adenomas, and medullary thyroid carcinomas [44].	Pentetreotide only binds with high affinity to the somatostatin receptor subtype SSTR2 with moderate affinity to SSTR3 and SSTR5 and not to SSTR1 and SSTR4 [30].

In-111 lanreotide (Somatuline)	This peptide is modified with DOTA and labeled with ^111^In in a similar way as octreotide [45, 46].	The high binding affinity of lanreotide to SSTR3 and SSTR4 makes it suitable to be used as a proper agent for visualization of certain tumors, such as intestinal adenocarcinomas which is not visualized by ^111^In-pentetreotide scintigraphy [47].	

^ 99m^Tc-labeled peptides			

Depreotide (NeoSpect)	Depreotide is a synthetic peptide that binds with high affinity to somatostatin receptors (SSTR) in normal as well as abnormal tissues it has been shown that. It accumulate in pulmonary nodules 1.5–2 hours following the i.v injection [48].	This agent is used to detect SSTR-bearing pulmonary masses in patients proven or suspected to have pulmonary lesions by CT and/or chest X-ray. Negative results with ^99m^Tc-Depreotide can exclude regional lymph node metastasis with a high degree of probability [49].	Labeling of the depreotide (cyclic decapeptide) with ^99m^Tc is performed by ligand exchange of intermediary ^99m^Tc-glucoheptonate [50].

Apcitide (AcuTect)	The kit vial contains a lyophilized mixture of depreotide, sodium glucoheptonate, stannous chloride, and sodium EDTA.	^99m^Tc-apticide binds to the GP IIb/IIIa receptors on activated platelets that are responsible for aggregation in forming the thrombi and, therefore, is used for the detection of acute deep vein thrombosis (DVT) in lower extremities [28].	
